# Prediction of infection in the emergency department—a machine learning model

**DOI:** 10.3389/frai.2026.1812692

**Published:** 2026-04-30

**Authors:** Sara N. Søgaard, Helene Skjøt-Arkil, Christian Backer Mogensen, Flemming Schønning Rosenvinge, Thomas Kronborg

**Affiliations:** 1 Department of Regional Health Research, University of Southern Denmark, Odense, Denmark; 2 Department of Emergency, University Hospital of Southern Denmark, Aabenraa, Denmark; 3 Department of Clinical Microbiology, Odense University Hospital, Odense, Denmark; 4 Research Unit of Clinical Microbiology, University of Southern Denmark, Odense, Denmark; 5 Department of Health Science and Technology, Aalborg University, Gistrup, Denmark

**Keywords:** clinical decision support tool, early diagnosis, emergency medicine, infection prediction, machine learning, antimicrobial stewardship

## Abstract

**Background:**

The integration of artificial intelligence (AI) into emergency medicine holds promise for enhancing early diagnosis and clinical decision-making. Traditional diagnostic approaches often rely on physician judgment or early warning scores that may delay identification of infections, especially when these tools prioritize outcomes such as mortality or ICU admission over early infection detection.

**Objective:**

This study aimed to identify the most informative predictors of infection at emergency department (ED) admission using machine learning (ML), and to develop a predictive model for infection among acutely admitted patients with suspected infection.

**Methods:**

Four ML algorithms were evaluated: Logistic Regression (LR), K-Nearest Neighbors (KNN), Decision Tree (DT), and Random Forest (RF). Sequential forward selection was applied to LR and KNN to optimize predictor input. DT and RF incorporated intrinsic feature selection. Model performance was assessed using sensitivity, specificity, positive/negative predictive value (PPV/NPV), accuracy, area under the ROC curve (AUC), average precision score (AP) with five-fold cross-validation.

**Results:**

RF model outperformed the other ML models, achieving a specificity of 75%, a PPV of 94%, and an accuracy of 80% while DT showed marginally higher sensitivity but poorer specificity. LR and KNN demonstrated intermediate performance. The RF model had the highest mean AUC of 84% and AP of 96%.

**Conclusion:**

RF model using readily available clinical variables—including C-reactive protein, leucocyte count, temperature, diastolic blood pressure and heart rate—can effectively predict infection at ED admission. This supports the potential of ML to enhance early infection detection and guide timely treatment in emergency settings.

## Highlights

- Random Forest model can predict infection at emergency department admission.
- Random forest model detects infection earlier than standard clinical scores.
- Key predictors: C-reactive protein, leucocyte count, temperature, blood pressure and heart rate.

## 1 Introduction

Artificial Intelligence (AI) integration into healthcare has advanced rapidly, with machine learning (ML) models transforming disease prediction and clinical decisions. Improved computational tools and large datasets have enabled sophisticated algorithms to analyze complex patient data. In emergency medicine, timely and accurate predictions are crucial for better patient outcomes, treatment and efficient resource use (Shafaf and Malek, 2019; Skjøt-Arkil et al., 2021).

Infections are a leading cause of admission in the Emergency Department (ED) (Statistics Denmark, 2022; Wang et al., 2017), and appropriate prescription and administration of antibiotics are crucial for minimizing the development of antibiotic resistance (Barnes et al., 2017; Zay Ya et al., 2023). To promote prudent and responsible use of antibiotics, it is essential to accurately identify the site of infection, thereby facilitating adherence to established antibiotic guidelines (May et al., 2013). In this context, a clear distinction between infection and more severe systemic responses such as sepsis is essential.

Sepsis is the body’s response to an infection leading to multiple organ failure. An infection is the presence and growth of pathogens. The distinction between sepsis prediction and infection prediction relies on the Sepsis-3 definition (Singer et al., 2016), in which sepsis requires the presence of organ dysfunction, typically represented clinically by an increase of 2 or more points in the Sequential Organ Failure Assessment (SOFA) score. In contrast, organ dysfunction is not required for the prediction or diagnosis of infection.

Considering this distinction, clinical tools have been developed to support early risk stratification in the ED, particularly with respect to identifying patients at risk of deterioration and/or sepsis. Early warning scores (EWS), such as qSOFA, NEWS2, MEWS or SIRS are widely used in the ED to assess the risk of sepsis, intensive care unit (ICU) admission or death, aiming to prevent adverse outcomes and enable early intervention (Gerry et al., 2020). However, focus here is on adverse outcomes and not the identification of patients with an infection that is not critical (Lam et al., 2024). EWS typically relies on clinical parameters such as temperature, blood oxygen saturation, and heart rate. However, their predictive performance has notable limitations (Hincapié-Osorno et al., 2024; Doğan et al., 2024).

One key challenge in diagnosing infections in the ED is the varied clinical presentations and the urgency of care (Caterino and Stevenson, 2012). Traditional diagnostic approaches often depend on physicians’ subjective judgment and EWS. Although these tools facilitate clinical assessment, they may inadvertently introduce diagnostic delays—for instance, by triaging patients to a lower acuity level than their actual condition warrants, or because their design prioritizes prediction of outcomes like mortality or ICU admission rather than early identification of infection. ML models offer a data-driven approach by integrating clinical variables, laboratory findings, and demographic data to uncover risk patterns that can remain undetected by conventional clinical judgment (Kareemi et al., 2021; Obermeyer and Emanuel, 2016).

When looking at ML models predicting infections, some ML models do not surpass the predictive accuracy of current clinical tools while others demonstrate substantially better predictive capabilities (van Boekel et al., 2024).

However, clinical adoption is challenged by the interpretability of ML models. Healthcare providers must be able to understand and trust the predictions made by these models in order to integrate them into their decision-making processes effectively (Holzinger et al., 2020). This emphasizes the need for a tool based on known and relevant factors. The aim of the present study was therefore to (1) utilize ML to identify the most informative predictors of infection upon ED admission, and (2) develop a model for predicting infections among patients acutely admitted to the ED with suspected infection. While models for predicting infection or bacteremia in ED populations exist, the majority of studies focus on sepsis or downstream outcomes such as ICU admission or mortality. To our knowledge, early diagnostic models specifically targeting infection within the first 4 h of ED presentation remain limited.

## 2 Methods

The study is reported according to Transparent Reporting of a multivariable prediction model for Individual Prognosis Or Diagnosis (TRIPOD) statement including update for artificial intelligence (The BMJ, 2024).

### 2.1 Data and participants

This study was a clinical predictive modeling study of data collected prospectively in a multicenter multifaceted study—the INDEED study (Trial IDs: NCT04661085, NCT04681963, NCT04667195, NCT04652167, NCT04686318, NCT04686292, NCT04651712, NCT04645030, NCT04651244), whose protocol has been previously published (Skjøt-Arkil et al., 2021). Patients in the study were admitted to the Emergency Departments at Odense University Hospital, Hospital Lillebaelt in Kolding, and Hospital Sønderjylland in Sønderborg and Aabenraa between March 1, 2021, and February 28, 2022.

The processing of data was authorized by the Region of Southern Denmark (approval no. 20/60508) in accordance with Article 30 of the EU General Data Protection Regulation. Ethical approval was obtained from the Regional Committee on Health Research Ethics for Southern Denmark (S-20200188). Furthermore, the study was registered with ClinicalTrials.gov (identifier NCT04651712) and conducted in adherence to the ethical principles outlined in the Declaration of Helsinki concerning medical research involving human participants.

### 2.2 Participants

The study included patients aged 18 years or older who were admitted to the ED suspected of having an infection and able to provide consent. Patients were excluded if they were directly transferred to intensive care, if participation could delay life-saving treatment, if they were admitted within the last 14 days, had a confirmed COVID-19 infection within the previous 14 days, or had severe immunodeficiency. Additional details on participant eligibility are available in the published protocol (Skjøt-Arkil et al., 2021). A total of 966 patients were included in the study, of whom 789 had a confirmed infectious diagnosis, while 177 had no verified infection. The final diagnosis was determined by an expert panel from each hospital consisting of a specialist in emergency medicine and a specialist in infectious medicine, both with extensive experience in acute infections. In total, eight experts were involved in the study. The diagnoses were based on chart reviews using all information obtained (including standard laboratory results and imaging) within the first week of admission. No checklists or diagnostic criteria were applied. Disagreements in the final diagnoses were resolved by the two experts in collaboration.

### 2.3 Data preparation, missing data and variables

The initial dataset included 858 variables extracted from medical records, encompassing symptoms, lifestyle factors, signs of disease severity, vital parameters, triage upon arrival, comorbidities, functional status, residence status, prior antibiotic prescriptions, and medical history. It also included laboratory results from biochemical blood tests, microbiological analysis of blood and sputum samples, and diagnostic imaging.

As the model was designed for early infection prediction, only factors available at the initial evaluation, defined as within the first 4 h of admission, including triage data and initial blood test results, were included. Variables with more than 10% missing data were excluded from the study. For variables with fewer missing data, which represented only 0.03% of the dataset, Multiple Imputation by Chained Equations (MICE) was applied prior to analysis and cross validation to address the missing values, allowing their inclusion in the analysis, resulting in a total of 132 variables for the model development. An overview of all included variables can be found in Appendix 1. Numerical variables were standardized to ensure consistent processing across several models. All data pre-processing and analyses were performed in python (version 3.7) with relevant packages.

### 2.4 Outcome

The study outcome of whether an infection was present or not, defined as a binary variable (yes or no), based on the expert panel diagnosis.

### 2.5 Analytical methods

We investigated four distinct machine learning models for the binary classification problem: Logistic Regression (LR), K-Nearest Neighbors (KNN), Decision Tree (DT), and Random Forest (RF). For each model, we reported sensitivity, specificity, negative predictive value (NPV), positive predictive value (PPV), accuracy, and the area under the receiver operating characteristic curve (AUC).

A sequential forward selection was employed to identify the most informative predictors for the LR and KNN models prior to training. This selection was conducted using the SequentialFeatureSelector function (library: mlxtend), with LR and KNN as the classifiers (library: scikit-learn). The five most informative predictors were reported.

For both the DT and RF models, forward selection was not applied, as it is inherently integrated into the model training process. In the DT model, the five predictors with the highest AUC were reported. In the RF model, the five most informative predictors were identified based on their importance factor (IF).

In addition to AUC, we also reported the Average Precision (AP) score derived from the precision-recall curve. While AUC provides a measure of overall discriminative ability, AP is particularly informative in imbalanced datasets, as it focuses on the model’s performance in correctly identifying positive cases.

### 2.6 Model training, validation and output

For the DT and RF models, hyperparameter tuning was performed using a grid search with 5-fold cross-validation on the data set.

Five-fold cross-validation was then used to train and validate each model, assessing their performance in identifying patients with infections using receiver operating characteristic (ROC) analysis. Performance metrics were averaged across the validation folds. A sensitivity threshold of 0.8 was applied. The optimal predictive model, incorporating the selected variables, was determined based on the highest overall mean AUC.

## 3 Results

### 3.1 Participants

The baseline characteristics of the included patients are presented in Table 1.

**Table 1:** Baseline characteristics of the included patients.

Characteristics	Infected (*n* = 789)	Not infected (*n* = 177)	All patients (*n* = 966)
Age in years, mean (SD)	69.2 (17.2)	65.8 (18.2)	68.6 (17.5)
Sex male, *n* (%)	439 (55.6)	82 (46.3)	521 (54.0)
Patient reported symptoms			
Chest pain, *n* (%)	135 (17.1)	38 (21.5)	173 (17.9)
Abdominal pain, *n* (%)	154 (19.5)	40 (22.6)	194 (20.1)
Fever feeling, *n* (%)	402 (51.0)	60 (33.9)	462 (47.8)
Fever chills, *n* (%)	380 (48.1)	50 (28.2)	430 (44.5)
Fever night sweat, *n* (%)	276 (35.0)	45 (25.4)	321 (33.2)
Measured fever, *n* (%)	291 (36.9)	36 (20.3)	327 (33.9)
Medication			
Polypharmacy (5 ≥ medication), *n* (%)	458 (58.1)	94 (53.1)	552 (57.1)
Vaccination status			
Covid vaccination, *n* (%)	634 (80.4)	132 (74.6)	766 (79.3)
Pneumococcal vaccination, *n* (%)	450 (57.0)	88 (49.7)	538 (55.7)
Influenza season vaccination, *n* (%)	540 (68.4)	102 (57.6)	642 (66.5)
Vital signs			
Heart Rate in beats/min, mean (SD)	91 (18)	87 (18)	90 (18)
Respiration frequency, n/min (SD)	20 (5)	19 (5)	20 (5)
Saturation, % (SD)	95 (4)	96 (3)	96 (3)
Systolic Blood Pressure, mmHg (SD)	132 (22)	135 (23)	133 (22)
Diastolic Blood Pressure, mmHg (SD)	74 (15)	78 (16)	75 (15)
Temperature, °C (SD)	37.6 (1.0)	37.0 (0.7)	37.5 (1.0)
GCS (Glascow Coma Score) value, (SD)	15 (0.3)	15 (0.4)	15 (0.3)
Clinical findings			
Crepitations on auscultation, *n* (%)	199 (25.2)	35 (19.8)	234 (24.2)
Ronchi on auscultation, *n* (%)	72 (9.1)	18 (10.2)	90 (9.3)
Prolonged expiration on auscultation, *n* (%)	34 (4.3)	8 (4.5)	42 (4.4)
Diminished breath sounds on auscultation, *n* (%)	20 (2.5)	2 (1.1)	22 (2.3)
Pain on abdominal palpation, *n* (%)	154 (19.5)	40 (22.6)	194 (20.1)
Blood samples			
Hemoglobin, mmol/L (SD)	7.9 (1.1)	8.0 (1.2)	7.9 (1.2)
Leucocytes, mia./L (SD)	12.9 (6.4)	9.6 (4.2)	12.3 (6.2)
C-reactive protein, mg/L (SD)	133.3 (101.9)	46.7 (65.8)	117.4 (101.9)
Creatinine, μmol/L (SD)	106.9 (84.9)	97.3 (82.9)	105.2 (84.5)
Natrium, mmol/L (SD)	136.2 (4.2)	137 (3.7)	136.5 (4.2)
eGFR, mL/min/1.73 m^2^ (SD)	61.2 (25.7)	67.2 (23.6)	62.3 (25.4)

SD, Standard deviation; eGFR, estimated Glomerular Filtration Rate.

### 3.2 Model development

The models were developed on data from 966 patients and based on 132 variables.

### 3.3 Model specification

In feature selection, several models selected common predictors, specifically C-reactive protein (CRP) and temperature. Individually, the LR model also selected chest pain, whereas the KNN model selected rheumatic disease and patient-reported fever. The DT model highlighted additional predictors including intermittent chest pain, urinary retention, other neurological diseases, cancer treatment, and tight chest pain. In contrast, the RF model selected a distinct set of predictors that are clinically measurable, namely leucocytes, heart rate and diastolic blood pressure. A detailed overview of the predictors for each model can be found in Table 2.

**Table 2:** Predictors from feature selection and AUC.

Model	Predictors	
Logistic regression	C-reactive protein	AUC 0.792
	Temperature	AUC 0.809
	Chest pain	AUC 0.814
	Oxygen saturation	AUC 0.818
	Sex	AUC 0.820
K nearest neighbors	C-reactive protein	AUC 0.767
	Temperature	AUC 0.800
	Rheumatic disease	AUC 0.808
	Patient reported fever	AUC 0.816
	Diabetes type 1	AUC 0.818
Decision tree	Tight chest pain	AUC 0.700
	Cancer treatment	AUC 0.690
	Other neurological disease	AUC 0.685
	Urinary retention	AUC 0.674
	Intermittent chest pain	AUC 0.653
Random forest	C-reactive protein	IF 0.227
	Leucocytes	IF 0.098
	Temperature	IF 0.059
	Diastolic blood pressure	IF 0.049
	Heart rate	IF 0.005

AUC, Area under the curve; IF, Importance Factor.

### 3.4 Model performance

The performance of the four models is detailed in Table 3.

**Table 3:** Model performance test to identify infection.

Model	Sensitivity, %	Specificity, %	Negative predictive value, %	Positive predictive value, %	Accuracy, %	Negative likelihood ratio	Positive likelihood ratio	Overall mean AUC for the model (CI 95%)
Logistic regression	82	65	43	92	79	0.28	2.32	0.80 (0.734–0.861)
K nearest neighbors	80	61	40	91	77	0.33	1.23	0.81 (0.740–0.861)
Decision tree	82	49	37	88	77	0.36	1.62	0.69 (0.606–0.777)
Random forest	81	75	45	94	80	0.26	3.17	0.84 (0.780–0.888)

CI, confidence interval.

Among the evaluated models, the RF model demonstrated the most balanced and robust performance, achieving the highest specificity, PPV, NPV, and overall accuracy. While the DT model showed slightly higher sensitivity, this came at the cost of substantially lower specificity. The LR and KNN models yielded intermediate results, although the LR model slightly outperformed the KNN model on all parameters despite overall AUC score.

**Figure 1:**
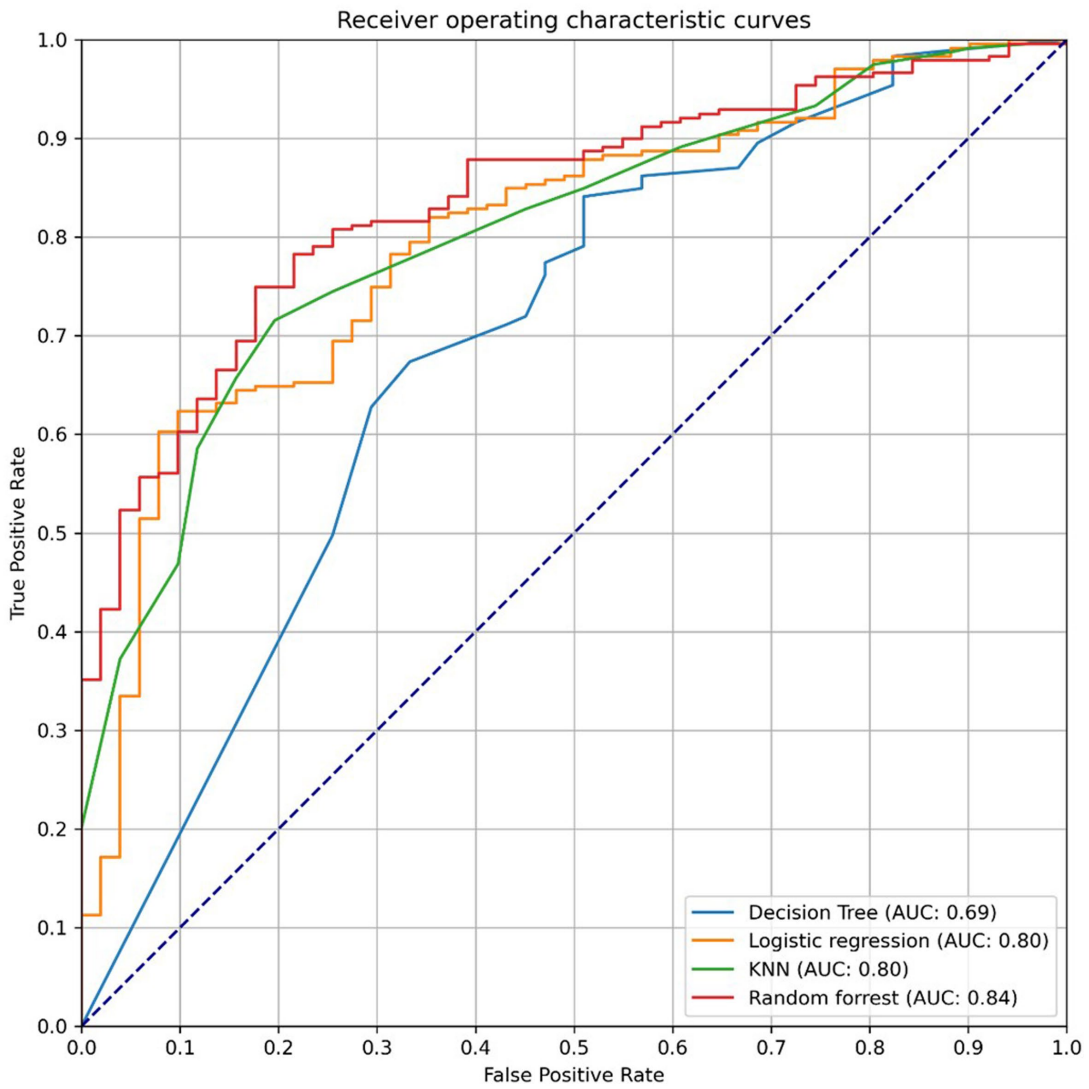
Receiver operating characteristics curve for the four models. AUC, Area under the receiver operating characteristics curve.

The best performing model, based on overall mean AUC score, was the RF model, as illustrated in Figure 1. The 95% confidence intervals for the AUC scores indicated that the discriminative performance of the DT model was significantly lower than that of the RF model, while the intervals for the LR and KNN models still overlapped with RF, suggesting no significant differences among these models.

In terms of likelihood ratios, the RF model demonstrated the strongest diagnostic utility, with the highest positive likelihood ratio (3.17) and the lowest negative likelihood ratio (0.26), suggesting a greater ability to both confirm and exclude infection. In contrast, the DT and KNN models exhibited weaker performance, particularly in their positive likelihood ratios (1.62 and 1.23, respectively), indicating more limited rule-in capability. The LR model showed moderate diagnostic value, with a positive likelihood ratio of 2.32 and a negative likelihood ratio of 0.28, indicating a balanced performance, though not as strong as that of the RF model.

**Figure 2:**
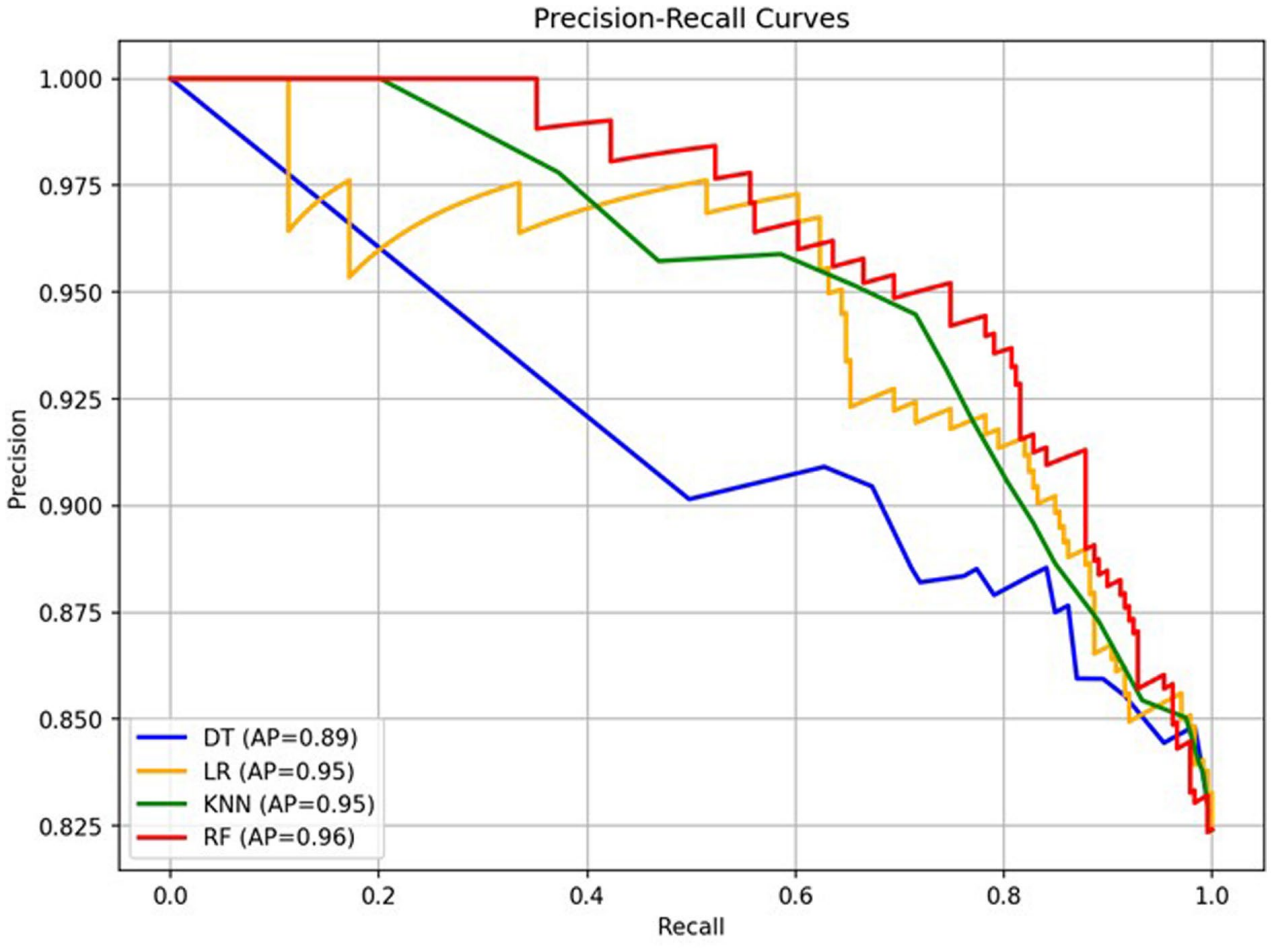
Precision-recall curve for the four models. AP, Average precision.

Figure 2 shows the precision recall curves for all four ML models. The RF model performs the best, achieving an AP of 0.96, the LR and KNN reaches an AP of 0.95 while the lowest AP was reached by the DT model (AP of 0.89).

## 4 Discussion

In this study, we evaluated four machine learning models for predicting infection in an ED population with suspected infection, and found that the RF model demonstrated the most consistent and robust performance. This was reflected not only in the highest mean AUC score of 84%, but also in its likelihood ratios of LR- 0.26 and LR + 3.17, and highest AP of 96%, suggesting a superior ability to both confirm and exclude infection. The model’s clinical relevance is further supported by its selection of the predictors CRP, leucocytes, temperature, diastolic blood pressure, and heart rate, which align well with established diagnostic practices. A high PPV of 94% underscores its reliability in confirming infection when predicted, while a specificity of 75% highlights its effectiveness in reducing false positives—an important consideration in the emergency department to prevent unnecessary treatments and investigations. It should be noted that these results, for all evaluated models, are based on a highly selected patient population predominantly consisting of individuals with infection, which may have influenced overall model performance and limits generalisability to a routine emergency department population. The high prevalence of infection in this cohort could potentially lead to overestimation of performance metrics. However, reporting AP alongside AUC thus provides a complementary perspective on model performance, highlighting the ability to maintain high precision across different levels of recall—despite the imbalanced data.

The RF model achieves the best performance, maintaining high precision across a wide range of recall values. This indicates a strong ability to correctly identify positive cases while minimizing false positives. Both LR and KNN had an AP of 95%, showing similarly high performance, with stable precision at relatively high recall levels.

In contrast, the DT model performs noticeably worse. Its precision curve declines more rapidly as recall increases, indicating that it produces more false positives when trying to capture more true positives. The lower AP value reflects this weaker overall performance of the model.

The KNN and LR models performed with AUC scores of 81 and 80% and PPVs of 91 and 92%, respectively. However, from a clinical perspective, the predictors identified by these models introduce a degree of uncertainty when aiming for consistent and reliable patient evaluation. For instance, variables such as chest pain, urinary retention, other neurological disease, cancer treatment, and rheumatic disease are highly heterogeneous and often non-specific. A symptom like chest pain, for example, can be difficult to verify objectively and may stem from a wide range of underlying conditions, many of which are non-infectious. This makes it challenging to uniformly interpret such predictors as indicators of infection across diverse patient presentations.

Compared to these models, the DT model performed notably worse, with a considerably lower overall AUC score and a specificity of only 53%. In addition, the clinical relevance of the predictors identified by the DT model appears limited in the context of infection, further reducing its potential utility in clinical decision-making.

Most EWS currently used in emergency departments were not developed to identify infection, but rather to predict outcomes such as mortality or ICU admission. A systematic review comparing the EWS qSOFA and CURB-65 across 19 studies found that the primary focus of these is outcome severity rather than detection of infection itself (Gelaidan et al., 2024). This highlights a fundamental limitation in their clinical utility when early infection identification is the goal. In recent years, ML has been studied as a prediction tool in pediatric populations—including for critical illness (Hwang and Lee, 2022), sepsis (Gomes et al., 2025) and bronchiolitis (Raita et al., 2020). Notably, temperature and respiratory rate are consistently highlighted as key prognostic variables in several of these studies, aligning with the findings of our best-performing model. While most studies focus on early identification of illness, some have specifically explored ML for predicting progression to sepsis rather than merely detecting infection (Liu et al., 2025; Zhang et al., 2021).

The RF model identified the most informative and predictive variables as general clinical parameters available early in the admission process. These parameters, such as vital signs, are routinely collected for nearly all patients in the ED, regardless of their condition. The reliance on general rather than specific biomarkers highlight the model’s potential to support clinicians by flagging patients at risk of infection early in their trajectory. This is consistent with previous findings showing that simple clinical measurements can contribute to the early identification of infection and sepsis (Lane et al., 2020). This capability could enhance timely diagnosis and enable earlier initiation of treatment, potentially improving outcomes by identifying cases of infection in an ED.

The dataset is unique in the way, that patient reported symptoms were prospectively systematically collected allongside clinical information, biological samples and diagnostic imaging. Further the process of data colletion was streamlined in questionaires and a research database to minimize the risk of bias.

## 5 Limitations

The development of this model is limited by selection bias, as the dataset includes only patients already suspected of having an infection. Data collection was further constrained to weekdays during daytime hours and required patients to provide both written and oral consent. As a result, the study population was highly selected, with an infection prevalence of 82%, which may lead to an overestimation of the model’s predictive ability in routine clinical settings.

Furthermore, the exclusion of patients unable to consent—such as those with impaired consciousness, language barriers, or cognitive deficits—means that certain clinically relevant subgroups were underrepresented. These patient groups are often among the most diagnostically complex and could particularly benefit from improved decision support.

To ensure broader applicability, future research should validate the model in more diverse and unselected emergency department populations, capturing the full spectrum of clinical presentations. Further research could also include more complex machine learning models.

## 6 Conclusion

A RF model based on initial clinical variables are promising in the prediction of infections in patients admitted to the ED with suspected infection. The most informative variables for the model were C-reactive protein, leucocytes, temperature, diastolic blood pressure and heart rate. These findings highlight the potential of machine learning for prediction of infection in the ED, based on well-known and available clinical markers, to improve early diagnosis and treatment strategies in clinical practice, as early diagnostic identification is crucial for enabling timely intervention, reducing disease progression, and improving patient outcomes.

## Data Availability

The data analyzed in this study is subject to the following licenses/restrictions: Due to Danish laws on personal data, data cannot be shared publicly. To request data, please contact the corresponding author for more information. This organization behind the INDEED study owns the data and can provide access to the final dataset. Requests to access these datasets should be directed to sns@rsyd.dk.
