# Supplementary material for: Prediction of infection in the emergency department—a machine learning model
Source: Front Artif Intell. 2026 Apr 30;9:1812692. doi: 10.3389/frai.2026.1812692 (PMC13171526; doi:10.3389/frai.2026.1812692)
Supplement: Supplementary file 1 [file Table_1.DOCX]

Supplementary material

Appendix 1:

| Id number |
| --- |
| Patient age |
| Patient sex |
| Medical record information |
| Triage information |
| Respirations frequence |
| Saturation |
| Heart rate |
| Systolic blood pressure |
| Diastolic blood pressure |
| Temperature |
| Glascow Coma Scale (GCS) |
| Blood samples |
| Hemoglobin |
| Leucocyttes |
| Kreatinin |
| Natrium |
| C-reactive protein (CRP) |
| Estimated glomerular filtration rate (eGFR) |
| Clinical findings |
| Catheter |
| Oxygen requirements |
| Abnormal Chest auscultation |
| Chest auscultation with crepitations |
| Chest auscultation with ronchi |
| Chest auscultation with reduced breath sounds |
| Chest auscultation with prolonged expiration |
| Chest auscultation with other abnormalities |
| Palpatory abdominal pain |
| Palpatory abdominal pain – left flank |
| Palpatory abdominal pain – right flank |
| Palpatory abdominal pain – supra pubic |
| Palpatory abdominal pain – diffuse |
| Palpatory abdominal pain – other location |
| Medication and vaccinations |
| Paracetamol |
| Non-Steroidal Anti-Inflammatory Drugs |
| Opioids |
| Gabapentin |
| Analgetics |
| Other medications |
| Polyfarmaci |
| Covid vaccination status |
| Pneumoccoc vaccination status |
| Influenza vaccination status |
| No current vaccination |
| Patient reported symptoms |
| Fever feeling |
| Fever chills |
| Fever night sweat |
| Measured fever |
| Chest pain – central |
| Chest pain – diffuse |
| Chest pain – right |
| Chest pain – left |
| Chest pain – other location |
| Chest pain – tightening |
| Chest pain – sharp |
| Chest pain – burning |
| Chest pain – murmur |
| Chest pain – discomfort |
| Chest pain – other type |
| Chest pain – intermittent |
| Chest pain – respiratory dependent |
| Chest pain – position dependent |
| Chest pain – activity dependent |
| Chest pain – radiation pain |
| Chest pain – other characteristic |
| Gastrointestinal pain – diffuse |
| Gastrointestinal pain – suprapubic |
| Gastrointestinal pain – epigastriel |
| Gastrointestinal pain – right flank |
| Gastrointestinal pain – left flank |
| Gastrointestinal pain – upper right quadrant |
| Gastrointestinal pain – lower right quadrant |
| Gastrointestinal pain – upper left quadrant |
| Gastrointestinal pain – lower left quadrant |
| Comorbidities |
| Neurological disease |
| Neurological cerebrovascular disease |
| Parkinson disease |
| Multiple scleroses |
| Dementia |
| Other neurological disease |
| Lung Disease |
| COPD |
| Asthma |
| Other pulmonic disease |
| Endocrinology disease |
| Diabetes type 1 |
| Diabetes type 2 |
| Osteoporosis |
| Other endocrinology disease |
| Nephrological disease |
| Chronic Kidney Disease (CKD) |
| Kidney stone |
| Incontinent |
| Urine retention |
| Urinary catheter |
| Clean Intermittent Catheterization (CIC) |
| Sterile Intermittent Catheterization (SIC) |
| Suprapubic catheter |
| Other nephrological disease |
| Cardiac disease |
| Ischemic heart disease (IHD) |
| Heart failure |
| Heart arrhythmia |
| Pericardial effusion |
| Deep venous thrombosis |
| Vascular insufficiency |
| Other cardiac disease |
| Gastrointestinal disease |
| Liver disease |
| Inflammatory Bowel Disease |
| PEG tube |
| Other gastrointestinal disease |
| Rheumatologic disease |
| Rheumatoid arthritis |
| Polymyalgia rheumatica |
| Gout |
| Other rheumatologic disease |
| Cancer diagnosis |
| Cancer diagnosis – Lung |
| Cancer diagnosis – urinary tract |
| Cancer diagnosis – gynecological or abdominal |
| Cancer diagnosis – oesophagus |
| Cancer diagnosis – Ear, nose, throat. |
| Cancer diagnosis – haematological |
| Cancer diagnosis – brain |
| Cancer diagnosis – breast |
| Cancer diagnosis – other site |
| Cancer treatment – chemo |
| Cancer treatment – radiation therapy |
| Cancer treatment – other type |
| Follow up |
| Intensive care admission within 30 days |
| Patient mortality |
| Infected patients |
